# Prehospital Trauma Care in Disasters and Other Mass Casualty Incidents – A Proposal for Hospital-Based Special Medical Response Teams

**DOI:** 10.7759/cureus.13657

**Published:** 2021-03-02

**Authors:** Michael Neeki, Stephen DuMontier, Jake Toy, Benjamin Archambeau, Eric Goralnick, Troy Pennington, Kenji Inaba, Rick Hammesfahr, David Wong, David S Plurad

**Affiliations:** 1 Emergency Medicine, California University of Science and Medicine, Colton, USA; 2 Emergency Medicine, Arrowhead Regional Medical Center, Colton, USA; 3 Emergency Medicine, Harbor University of California Los Angeles Medical Center, Torrance, USA; 4 Surgery, Brigham and Women’s Hospital, Boston, USA; 5 Surgery, University of Southern California, Los Angeles, USA; 6 Tactical Emergency Support Team, Marietta Police and Fire Department, Marietta, USA; 7 Surgery, Arrowhead Regional Medical Center, Colton, USA; 8 Surgery, California University of Science and Medicine, Colton, USA; 9 Department of Surgery, Riverside Community Hospital, Riverside, USA

**Keywords:** disaster, mass casualty incident, triage, emergency medical services

## Abstract

Current mass casualty incident (MCI) response in the United States calls for rapid deployment of first responders, such as law enforcement, fire, and emergency medical services personnel, to the incident and simultaneous activation of trauma center disaster protocols. Past investigations demonstrated that the incorporation of advanced trauma-trained physicians and paramedics into prehospital teams resulted in improved mortality during routine emergency medical care in Europe and in the combat setting. To date, limited research exists on the incorporation of advanced trauma-trained physicians and paramedics into prehospital teams for civilian MCIs.

We proposed the concept of Special Medical Response Teams, which would rapidly deploy advanced trauma-trained physicians and paramedics to deliver a higher level of medical and surgical care in the prehospital setting during civilian mass casualty incidents.

## Introduction

Mass casualty incidents (MCIs) such as local, regional, or national disasters, as well as mass shootings and bombings in the United States, are a persistent threat that mandates healthcare system preparedness [1]. The Federal Emergency Management Agency (FEMA) defines an MCI as any event, planned or unplanned, that results in the need to provide medical care to patients outside of traditional hospital settings [1,2]. A major challenge associated with these events is the triage and treatment of a large number of critical casualties in a short time period [3]. In light of the increased frequency of mass shootings in the civilian sector, public initiatives have been established in an effort to reduce the preventable loss of life. Two main examples were the Hartford Consensus and Tactical Emergency Casualty Care (TECC) guidelines.

The Hartford Consensus is a collaboration of more than 35 public and private entities that was conceived shortly after the 2012 Sandy Hook Elementary School tragedy. Trauma care guidelines were developed to minimize loss of life in an active shooter event and MCI in the civilian arena. The Hartford Consensus called for more rapid on-scene interventions addressing preventable deaths through an integrated response involving existing emergency medical techniques and equipment for law enforcement, fire, emergency medical services (EMS), and lay-persons. The Hartford Consensus further provided recommendations for critical actions to enable timely access through tiered response zones determined by proximity [4].

Experts also advocated for increased implementation of Tactical Emergency Casualty Care (TECC) training for first responders. TECC is the civilian adaptation of Tactical Combat Casualty Care (TCCC) utilized by the US military, emphasizing early hemorrhage control in addition to the immediate correction of acute causes of death such as tension pneumothorax and airway obstruction [5-7]. Integration of military responses to trauma MCIs into the civilian sector has been strongly advocated [8].

Analyses of civilian intentional MCIs have noted significant differences in injury patterns compared to those sustained during combat-related trauma [9-11]. For example, civilian shooting victims exhibit fewer extremity injuries and sustain more severe injuries to the head, chest, and upper back, likely due to lack of body armor [9-11]. Civilian blast victims exhibit more severe injuries than conventional trauma victims, exemplified by the multidimensional blast injury patterns that often require specialized care teams [11].

Current prehospital emergency response in the United States calls for rapid deployment of law enforcement, fire department, and EMS personnel to the scene and simultaneous activation of trauma center disaster protocols. No known existing literature investigates the mortality benefit of the rapid deployment of advanced trauma-trained physicians and paramedics to MCIs. Past investigations, however, have demonstrated that the incorporation of specialized physicians into prehospital teams may result in improved mortality outcomes during routine emergency medical and trauma care [12-15]. Physician presence has proven beneficial in situations where there are critically injured trauma patients requiring advanced medical interventions [14-16]. Additionally, advanced trauma training provided to combat medics showed similar improvements in mortality outcomes [17].

Here, we evaluate the current models of civilian medical response to MCIs or disaster situations. Furthermore, we introduce the concept of Special Medical Response Teams (SMRT) with the rapid deployment of medical support capabilities in response to civilian MCIs. SMRT would act as a scalable adjunct to current prehospital emergency care and consist of advanced trauma-trained physicians, trauma nurses, and paramedics. SMRT seeks to address two key issues associated with modern MCIs: increased injury severity and the importance of accurate triage. The mission is to improve patient outcomes by initiating early damage control and lifesaving resuscitation measures led by advanced trauma-trained teams in the prehospital setting.

Issues specific to mass casualty incidents

Active Shooter Event Injury Profiles

Injury profiles from high-powered gunshots and detonated blasts are often complex, with massive hemorrhage as the leading cause of preventable death [18]. A recent analysis of 12 United States civilian mass shooting events over the past 30 years examined the prevalence of common injury patterns. Overall, 58% of victims sustained gunshot wounds to the head and chest, while 20% sustained extremity wounds. Seven percent of overall fatalities were retrospectively determined to have had potentially survivable injuries. The most common injuries were gunshot wounds to the chest. Pathology reports that lacked devastating traumatic pathology at autopsy lead the authors to suggest that these victims likely died due to tension pneumothorax or other treatable respiratory impairment [10].

Injuries sustained in civilian, active shooter incidents have further been noted to be vastly different from combat injuries [10]. Head and torso injuries are less common in the combat theater (48% vs. 72% in civilian), while exsanguinating extremity injuries are more common (52-64% vs. 20% in civilian). In the combat theater, over 15% of deaths were potentially survivable, with the majority resulting from exsanguinating hemorrhage, as compared to the previously noted study citing 7% of civilian deaths that were preventable [10,19,20].

Blast Injury Profile

Civilian blast victims have been observed to have more severe injuries than those noted in combat-focused studies. While 10% of non-terrorist trauma victims may present with an injury severity score (ISS) ≥16, this proportion was significantly higher among terrorist blast victims (close to 29%) [11]. Blast victims presented four times more frequently with a Glasgow Coma Scale (GCS) of less than five than conventional trauma victims, reflecting an increased injury severity and complexity [11]. Additionally, blast victims were twice as likely to present with hemodynamic instability [11]. MCIs have created a new type of civilian wounding pattern with a predominance of the head, chest, and abdominal injuries. In response, our strategy in preparation and management of these injury patterns must also evolve.

Over-Triage During Mass Casualty Incidents and Disasters

During MCIs, efficient triage is crucial for effectively treating large numbers of casualties. Determining which patients may benefit from lifesaving intervention in the field or from immediate transport based on resource availability impacts overall patient survival [21]. This is true with scenarios involving both over- and under-triage of patients. Over-triage can allocate resources to patients that do not require them, limiting resource availability to others who may benefit more. On the other hand, under-triage can result in definitive or life-saving care being delayed past a patient’s survivability window. In this regard, there are well-established precedents for deploying forward physicians to help triage patients in other EMS infrastructures and MCI response protocols in other regions of the world [22].

Analysis of the prehospital response to the London bombings on July 7th, 2005, measured the over-triage rates among the London Helicopter Emergency Medical Service (HEMS), which consists of advanced-trained physicians and paramedical staff, in comparison to the standard ambulance service and medically trained bystanders. Investigators reported significantly lower over-triage rates by physician-paramedic staffed London-HEMS teams of 35%, compared to average over-triage rates of 67% from other previous events around the world [22]. The same study reported a lower-than-expected critical mortality rate of 15% [22].

By comparison, during the World Trade Center (WTC) attacks on September 11th, 2001, the over-triage rates were 70% [22]. In this case, triage was primarily conducted by the fire department and EMS first responders. Critical mortality was 38% during this incident [23]. This difference in over-triage and critical mortality between the London and WTC attacks may be due in part to London’s major incident plan that requires physicians to be present at the scene to assist with triage, thereby reducing patient surges at receiving institutions.

## Materials and methods

Current models to manage MCIs

United States EMS Model

The North American EMS model emphasizes rapid transport of a casualty to the hospital with minimal care in the field, a model known as “Scoop and Run” [24]. In the United States, prehospital medical care is routinely provided by emergency medicine technicians (EMTs) with certifications ranging from basic (EMT-B) to paramedic (EMT-P). Scope of practice for paramedics varies regionally; however, most EMTs can perform endotracheal intubation (ETI), establish intravenous (IV) access, and administer selected pharmacological agents [24]. These teams are often dispatched without the aid of physicians and irrespective of patient acuity with instructions to minimize scene time and interventions [24,25].

Though controversial in existing literature, “Scoop and Run” is an effective method in urban environments with few victims, where short transports to more definitive care may negate the benefit of field interventions [24]. One study further suggests that non-EMS “Scoop and Run” without onboard medical care (e.g., by police) may be effective in selected cases such as penetrating trauma [26]. However, “Scoop and Run” may not be suited for MCIs in which multiple casualties must be managed in a short time [24]. In these cases, where transport may be delayed or impossible due to entrapment, isolation, or overload of receiving facilities, it may be most effective to provide advanced clinical management in the field or en route to definitive care in conjunction with effective triage and distribution of the critically injured to available hospital resources.

European EMS Model

The European model integrates the delivery of advanced medical management and interventions at the scene, a model aptly known as “Stay and Play.” Prehospital care teams are led by advanced trauma-trained physicians and supported by nurses and paramedics. In many non-critical cases, the patient may be effectively managed in the field and not require transportation to a higher level of care at all [25]. For critically injured patients, advanced medical management including ETI, cricothyrotomy, escharotomy, needle and chest tube thoracostomy, thoracotomy, blood transfusions, resuscitative endovascular balloon occlusion (REBOA), and extracorporeal membrane oxygenation (ECMO) may be initiated in the prehospital setting. Of note, however, based on a study by Hass and Nathens, performing advanced interventions in the field may prolong on-scene time and possibly result in negative patient outcomes [24].

During an MCI or disaster when “Scoop and Run” may not be feasible due to limited transport, “Stay and Play” may be the superior model due to its emphasis on advanced prehospital medical management by trauma-trained physicians and paramedics. In these situations, the United States emergency medical response system may benefit from direct medical control in the field to aid in triage, transport decision-making, and lifesaving prehospital interventions.

## Results

New proposed model

*Special Medical Response Teams (SMRT) - an Expansion of the Current Hospital Emergency Response Team/Training (HERT)* *Model*

Currently, in the United States, Hospital Emergency Response Team/Training (HERT) typically refers to a hospital’s plan to internally organize and respond to an MCI or disaster. Some institutions in Southern California, however, have successfully launched a team of physicians and nurses by the same name in support of on-scene firefighters and paramedics for advanced field resuscitation, often in life-over-limb situations requiring field amputations. Of the cases described in the literature, an enhanced hospital-based medical team led by a trauma surgeon and accompanied by a senior emergency medicine resident and a trauma nurse were able to implement advanced resuscitation on scene while firefighters worked to free a motorist that had been entrapped for over two hours. Because of the efforts of this enhanced care team (ECT), the motorist survived [27].

To complement emergency medical protocols for existing ECTs, we recommend the implementation of Special Medical Response Teams (SMRT) with rapid deployment capabilities. SMRT would incorporate advanced, trauma-trained physicians and nurses with selected paramedics from local agencies into prehospital teams with the ability to provide advanced on-scene damage control resuscitation and support. The main goal of SMRT would be to enhance survivability in the prehospital setting. This would be achieved through the initiation of advanced medical care in the field, assistance with triage, and acting as an adjunct to the development and training of integrated medical preparedness amongst first responders (e.g., law enforcement, fire department, community members).

The composition of SMRT would potentially include a trauma surgeon, an emergency medicine physician, trauma nurse(s), and paramedic(s) trained in advanced trauma management. All members of the team would be required to have and maintain certifications in trauma life support (i.e., Advanced Trauma Life Support (ATLS) for physicians or Trauma Nursing Core Course for nurses).

## Discussion

Though “Scoop and Run” has been accepted as the most effective management during MCIs traditionally, the evolving nature of MCIs in the current era yields the potential for increased scene time where an SMRT team could make a critical difference. The spectrum for response would include active shooter events, as well as intentional and unintentional MCIs, where advanced medical care can be provided in the field when rapid transport is not feasible. More specifically, SMRT would assist in events with multiple victims with potentially increased prehospital time. These may further include incidents involving a single victim with an anticipated prolonged extrication or incidents involving victim(s) in rural settings far removed from trauma centers. It may further include events involving chemical, biological, or radioactive agents with anticipated victim decontamination, incapacitation of regional hospitals, or large-scale destruction in a city center. Such a response would reduce unnecessary communication between field and base.

SMRT could assist in rapid triage and resuscitation procedures en route to the hospital in patients who may otherwise have a low chance of survival without early advanced intervention. Control of thoracic and abdominal hemorrhages represent specific areas in which SMRT can provide emergent definitive care that is out of the scope of current EMS providers’ practice. SMRT deployment must not be done at the expense of resource availability from receiving trauma centers. Only selective regional trauma centers with adequate resources should develop an SMRT program.

The capabilities of SMRT would be varied depending on regional system needs and resources and serve as a mode to deploy the most advanced medical technology into the field, expanding on practices seen in other civilian trauma systems currently implemented around the world. SMRT would serve as an adjunct to first responders, providing on-scene resuscitation, extremity hemorrhage control, monitoring of patient(s), pain management, sedation, and advanced airway support. In addition, SMRT could perform procedures outside the scope of practice of paramedics or non-routine procedures with limited additional training or maintenance of skills. These would include blood transfusion and amputation of entrapped limb(s) if necessary. For severely unstable patients with potentially survivable injuries, advanced resuscitation adjuncts such as thoracotomy, escharotomy, ECMO cannulation, or REBOA may also be considered on-scene or en-route [15,28-30]. These procedures are most effective when performed quickly.

The team would work in conjunction with established Incident Command models to determine where patients should be transported to reduce over-triage in a high patient volume incident. The team would operate under the Incident Command System (ICS) and be activated by an MCI incident commander (IC), operating in a designated area where they can be most effective. Alternatively, the SMRT team could be deployed in mass gathering events in anticipation of its utilization, such as large races or concerts with hundreds or thousands of participants.

## Conclusions

Each MCI is unique and often requires evolving preparation and training to adequately equip providers to handle triage, resuscitation, and stabilization appropriately. A significant body of evidence supports prehospital physician presence and the benefits of paramedics with advanced training during routine emergency care. The SMRT model may offer a solution toward improving MCI survivability and overall public safety during disasters. The future of SMRT rests in the integration of its team members into a cohesive EMS system. By introducing hospital-based physicians and nurses to the prehospital setting, and paramedics to advanced trauma techniques in the hospital, SMRT teams have the potential to significantly improve disability and mortality rates related to MCIs or disasters. The combination of paramedic skills in the prehospital arena and the clinical expertise from emergency and trauma-trained practicing physicians will improve prehospital care globally, especially in mass casualty or similar situations where it is most important.
